# Automating data extraction in systematic reviews: a systematic review

**DOI:** 10.1186/s13643-015-0066-7

**Published:** 2015-06-15

**Authors:** Siddhartha R. Jonnalagadda, Pawan Goyal, Mark D. Huffman

**Affiliations:** 1 Division of Health and Biomedical Informatics, Department of Preventive Medicine, Northwestern University Feinberg School of Medicine, 750 North Lake Shore Drive, 11th Floor, Chicago, IL 60611 USA; 2 Department of Computer Science and Engineering, Indian Institute of Technology, Kharagpur, 721302 West Bengal India; 3 Department of Preventive Medicine, Northwestern University Feinberg School of Medicine, Chicago, USA

## Abstract

**Background:**

Automation of the parts of systematic review process, specifically the data extraction step, may be an important strategy to reduce the time necessary to complete a systematic review. However, the state of the science of automatically extracting data elements from full texts has not been well described. This paper performs a systematic review of published and unpublished methods to automate data extraction for systematic reviews.

**Methods:**

We systematically searched PubMed, IEEEXplore, and ACM Digital Library to identify potentially relevant articles. We included reports that met the following criteria: 1) methods or results section described what entities were or need to be extracted, and 2) at least one entity was automatically extracted with evaluation results that were presented for that entity. We also reviewed the citations from included reports.

**Results:**

Out of a total of 1190 unique citations that met our search criteria, we found 26 published reports describing automatic extraction of at least one of more than 52 potential data elements used in systematic reviews. For 25 (48 %) of the data elements used in systematic reviews, there were attempts from various researchers to extract information automatically from the publication text. Out of these, 14 (27 %) data elements were completely extracted, but the highest number of data elements extracted automatically by a single study was 7. Most of the data elements were extracted with F-scores (a mean of sensitivity and positive predictive value) of over 70 %.

**Conclusions:**

We found no unified information extraction framework tailored to the systematic review process, and published reports focused on a limited (1–7) number of data elements. Biomedical natural language processing techniques have not been fully utilized to fully or even partially automate the data extraction step of systematic reviews.

## Background

Systematic reviews identify, assess, synthesize, and interpret published and unpublished evidence, which improves decision-making for clinicians, patients, policymakers, and other stakeholders [1]. Systematic reviews also identify research gaps to develop new research ideas. The steps to conduct a systematic review [1–3] are:

1. Define the review question and develop criteria for including studies
2. Search for studies addressing the review question
3. Select studies that meet criteria for inclusion in the review
4. Extract data from included studies
5. Assess the risk of bias in the included studies, by appraising them critically
6. Where appropriate, analyze the included data by undertaking meta-analyses
7. Address reporting biases

Despite their widely acknowledged usefulness [4], the process of systematic review, specifically the data extraction step (step 4), can be time-consuming. In fact, it typically takes 2.5–6.5 years for a primary study publication to be included and published in a new systematic review [5]. Further, within 2 years of the publication of systematic reviews, 23 % are out of date because they have not incorporated new evidence that might change the systematic review’s primary results [6].

Natural language processing (NLP), including text mining, involves information extraction, which is the discovery by computer of new, previously unfound information by automatically extracting information from different written resources [7]. Information extraction primarily constitutes concept extraction, also known as named entity recognition, and relation extraction, also known as association extraction. NLP handles written text at level of documents, words, grammar, meaning, and context. NLP techniques have been used to automate extraction of genomic and clinical information from biomedical literature. Similarly, automation of the data extraction step of the systematic review process through NLP may be one strategy to reduce the time necessary to complete and update a systematic review. The data extraction step is one of the most time-consuming steps of a systematic review. Automating or even semi-automating this step could substantially decrease the time taken to complete systematic reviews and thus decrease the time lag for research evidence to be translated into clinical practice. Despite these potential gains from NLP, the state of the science of automating data extraction has not been well described.

To date, there is limited knowledge and methods on how to automate the data extraction phase of the systematic reviews, despite being one of the most time-consuming steps. To address this gap in knowledge, we sought to perform a systematic review of methods to automate the data extraction component of the systematic review process.

## Methods

Our methodology was based on the Standards for Systematic Reviews set by the Institute of Medicine [8]. We conducted our study procedures as detailed below with input from the Cochrane Heart Group US Satellite.

### Eligibility criteria

We included a report that met the following criteria: 1) the methods or results section describes what entities were or needed to be extracted, and 2) at least one entity was automatically extracted with evaluation results that were presented for that entity.

We excluded a report that met any of the following criteria: 1) the methods were not applied to the data extraction step of a systematic review; 2) the report was an editorial, commentary, or other non-original research report; or 3) there was no evaluation component.

### Information sources and searches

For collecting the initial set of articles for our review, we developed search strategies with the help of the Cochrane Heart Group US Satellite, which includes systematic reviewers and a medical librarian. We refined these strategies using relevant citations from related papers. We searched three datasets: PubMed, IEEExplore, and ACM digital library, and our searches were limited between January 1, 2000 and January 6, 2015 (see Appendix1). We restricted our search to these dates because biomedical information extraction algorithms prior to 2000 are unlikely to be accurate enough to be used for systematic reviews.

We retrieved articles that dealt with the extraction of various data elements, defined as categories of data that pertained to any information about or deriving from a study, including details of methods, participants, setting, context, interventions, outcomes, results, publications, and investigators [1] from included study reports. After we retrieved the initial set of reports from the search results, we then evaluated reports included in the references of these reports. We also sought expert opinion for additional relevant citations.

### Study selection

We first de-duplicated the retrieve citations. For calibration and refinement of the inclusion and exclusion criteria, 100 citations were randomly selected and independently reviewed by a two authors (SRJ and PG). Disagreements were resolved by consensus with a third author (MH). In a second round, another set of 100 randomly selected abstracts was independently reviewed by two study authors (SRJ and PG), whereby we achieved a strong level of agreement (kappa = 0.97). Given the high level of agreement, the remaining studies were reviewed only by one author (PG). In this phase, we identified reports as “not relevant” or “potentially relevant”.

Two authors (PG and SRJ) independently reviewed the full text of all citations (*N* = 74) that were identified as “potentially relevant”. We classified included reports into various categories based on the particular data element that they attempted to extract from the original, scientific articles. Example of these data elements might be overall evidence, specific interventions, among others (Table 1). We resolved disagreements between the two reviewers through consensus with a third author (MDH).

**Table 1:** Data elements, category, sources and existing automation work

Data element	Category	Included in standards	Published method to extract?
Total number of participants	Participants	Cochrane, PICO, PECODR, PIBOSO, STARD	Yes [12, 13, 16–20, 23, 24, 28–30, 32, 39]
Settings	Participants	Cochrane, CONSORT, STARD	No
Diagnostic criteria	Participants	Cochrane, STARD	No
Age	Participants	Cochrane, STARD	Yes [24, 29, 39, 41]
Sex	Participants	Cochrane, STARD	Yes [24, 29, 41]
Country	Participants	Cochrane	Yes [24, 39]
Co-morbidity	Participants	Cochrane, STARD	Yes [21]
Socio-demographics	Participants	Cochrane, STARD	No
Spectrum of presenting symptoms, current treatments, recruitment centers	Participants	STARD	Yes [21, 24, 28, 29, 32, 41]
Ethnicity	Participants	Cochrane	Yes [41]
Date of study	Participants	Cochrane	Yes [39]
Date of recruitment and follow-up	Participants	CONSORT, STARD	No
Participant sampling	Participants	STARD	No
Total number of intervention groups	Intervention	Cochrane	Yes [34, 35]
Specific intervention	Intervention	Cochrane, PICO, PIBOSO, PECODR	Yes [12, 13, 16–20, 22, 24, 28, 34, 39, 40]
Intervention details (sufficient for replication, if feasible)	Intervention	Cochrane, CONSORT	Yes [36]
Integrity of intervention	Intervention	Cochrane	No
Outcomes and time points (i) collected; (ii) reported	Outcomes	Cochrane, CONSORT, PICO, PECODR, PIBOSO	Yes [12, 13, 16–20, 24, 25, 28, 34–36, 40]
Outcome definition (with diagnostic criteria if relevant)	Outcomes	Cochrane	No
Unit of measurement (if relevant)	Outcomes	Cochrane	No
For scales: upper and lower limits, and whether high or low score is good	Outcomes	Cochrane	No
Comparison	Comparisons	PICO, PECODR	Yes [12, 16, 22, 23]
Sample size	Results	Cochrane, CONSORT	Yes [36, 40]
Missing participants	Results	Cochrane	No
Summary data for each intervention group (e.g. 2 × 2 table for dichotomous data; means and SDs for continuous data)	Results	Cochrane, PECODR, STARD	No
Estimate of effect with confidence interval; *P* value	Results	Cochrane	No
Subgroup analyses	Results	Cochrane	No
Adverse events and side effects for each study group	Results	CONSORT, STARD	No
Overall evidence	Interpretation	CONSORT	Yes [26, 42]
Generalizability: external validity of trial findings	Interpretation	CONSORT	Yes [25]
Research questions and hypotheses	Objectives	CONSORT, PECODR, PIBOSO, STARD	Yes [24, 25]
Reference standard and its rationale	Method	STARD	No
Technical specifications of material and methods involved including how and when measurements were taken, and/or cite references for index tests and reference standard	Method	STARD	No
Study design	Method	Cochrane, PIBOSO	Yes [13, 18, 20, 24]
Total study duration	Method	Cochrane, PECODR	Yes [12, 29, 40]
Sequence generation	Method	Cochrane	Yes [27]
Allocation sequence concealment	Method	Cochrane	Yes [27]
Blinding	Method	Cochrane, CONSORT, STARD	Yes [27]
Methods used to generate random allocation sequence, implementation	Method	CONSORT, STARD	Yes [25]
Other concerns about bias	Method	Cochrane	No
Methods used to compare groups for primary outcomes and for additional analyses	Method	CONSORT, STARD	No
Methods for calculating test reproducibility	Method	STARD	No
Definition and rationale for the units, cutoffs and/or categories of the results of the index tests and reference standard	Method	STARD	No
Number, training, and expertise of the persons executing and reading the index tests and the reference standard	Method	STARD	No
Participant flow: flow of participants through each stage: randomly assigned, received intended treatment, completed study, analyzed for primary outcome, inclusion and exclusion criteria	Method	CONSORT	Yes [36, 37, 40]
Funding source	Miscellaneous	Cochrane	No
Key conclusions of the study authors	Miscellaneous	Cochrane	Yes [26]
Clinical applicability of the study findings	Miscellaneous	STARD	No
Miscellaneous comments from the study authors	Miscellaneous	Cochrane	No
References to other relevant studies	Miscellaneous	Cochrane	No
Correspondence required	Miscellaneous	Cochrane	No
Miscellaneous comments by the review authors	Miscellaneous	Cochrane	No

### Data collection process

Two authors (PG and SRJ) independently reviewed the included articles to extract data, such as the particular entity automatically extracted by the study, algorithm or technique used, and evaluation results into a data abstraction spreadsheet. We resolved disagreements through consensus with a third author (MDH).

### Data items

We reviewed the Cochrane Handbook for Systematic Reviews [1], the CONsolidated Standards Of Reporting Trials (CONSORT) [9] statement, the Standards for Reporting of Diagnostic Accuracy (STARD) initiative [10], and PICO [11], PECODR [12], and PIBOSO [13] frameworks to obtain the data elements to be considered. PICO stands for Population, Intervention, Comparison, Outcomes; PECODR stands for Patient-Population-Problem, Exposure-Intervention, Comparison, Outcome, Duration and Results; and PIBOSO stands for Population, Intervention, Background, Outcome, Study Design, Other.

### Data synthesis and analysis

Because of the large variation in study methods and measurements, a meta-analysis of methodological features and contextual factors associated with the frequency of data extraction methods was not possible. We therefore present a narrative synthesis of our findings. We did not thoroughly assess risk of bias, including reporting bias, for these reports because the study designs did not match domains evaluated in commonly used instruments such as the Cochrane Risk of Bias tool [1] or QUADAS-2 instrument used for systematic reviews of randomized trials and diagnostic test accuracy studies, respectively [14].

## Results

### Study selection

**Fig. 1:**
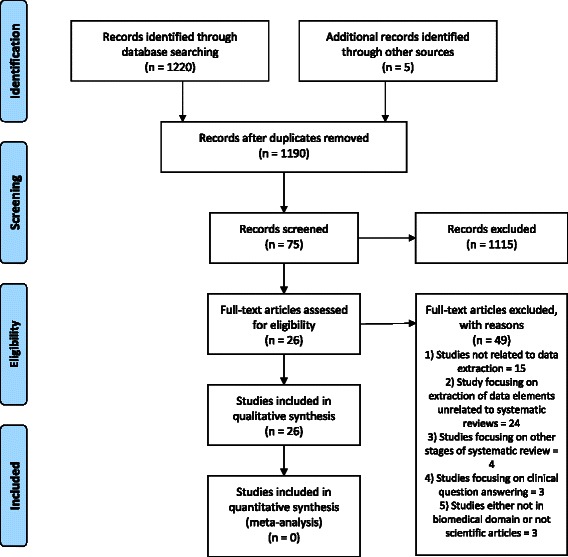
Process of screening the articles to be included for this systematic review

Of 1190 unique citations retrieved, we selected 75 reports for full-text screening, and we included 26 articles that met our inclusion criteria (Fig. 1). Agreement on abstract and full-text screening was 0.97 and 1.00.

### Study characteristics

Table 1 provides a list of items to be considered in the data extraction process based on the Cochrane Handbook (Appendix 2) [1], CONSORT statement [9], STARD initiative [10], and PICO [11], PECODR [12], and PIBOSO [13] frameworks. We provide the major group for each field and report which standard focused on that field. Finally, we report whether there was a published method to extract that field. Table 1 also identifies the data elements relevant to systematic review process categorized by their domain and the standard from which the element was adopted and was associated with existing automation methods, where present.

### Results of individual studies

Table 2 summarizes the existing information extraction studies. For each study, the table provides the citation to the study (study: column 1), data elements that the study focused on (extracted elements: column 2), dataset used by the study (dataset: column 3), algorithm and methods used for extraction (method: column 4), whether the study extracted only the sentence containing the data elements, full concept or neither of these (sentence/concept/neither: column 5), whether the extraction was done from full-text or abstracts (full text/abstract: column 6) and the main accuracy results reported by the system (results: column 7). The studies are arranged by increasing complexity by ordering studies that classified sentences before those that extracted the concepts and ordering studies that extracted data from abstracts before those that extracted data from full-text reports.

**Table 2:** A summary of included extraction methods and their evaluation

Study	Extracted elements	Dataset	Method	Sentence/Concept/Neither	Full text (F)/Abstract (A)	Results
Dawes et al. (2007) [12]	PECODR	20 evidence-based medicine journal synopses (759 extracts from the corresponding PubMed abstracts)	Proposed potential lexical patterns and assessed using NVIvo software	Neither	Abstract	Agreement among the annotators was 86.6 and 85 %, which rose up to 98.4 and 96.9 % after consensus. No automated system.
Kim et al. (2011) [13]	PIBOSO	1000 medical abstracts (PIBOSO corpus)	Conditional random fields with various features based on lexical, semantic, structural and sequential information	Sentence	Abstract	Micro-averaged F-scores on structured and unstructured: 80.9 and 66.9 %, 63.1 % on an external dataset
Boudin et al. (2010) [16]	PICO (I and C were combined together)	26,000 abstracts from PubMed, first sentences from the structured abstract	Combination of multiple supervised classification algorithms: random forests (RF), naive Bayes (NB), support vector machines (SVM), and multi-layer perceptron (MLP)	Sentence	Abstract	F-score of 86.3 % for P, 67 % for I (and C), and 56.3 % for O
Huang et al. (2011) [17]	PICO (except C)	23,472 sentences from the structured abstracts	naïve Bayes	Sentence	Abstract	F-measure of 0.91 for patient/problem, 0.75 for intervention, and 0.88 for outcome
Verbeke et al. (2012) [18]	PIBOSO	PIBOSO corpus	Statistical relational learning with kernels, kLog	Sentence	Abstract	Micro-averaged F of 84.29 % on structured abstracts and 67.14 % on unstructured abstracts
Huang et al. (2013) [19]	PICO (except C)	19,854 structured abstracts of randomized controlled trials	First sentence of the section or all sentences in the section, NB classifier	Sentence	Abstract	First sentence of the section: F-scores for P: 0.74, I: 0.66, and O: 0.73
						All sentences in the section: F-scores for P: 0.73, I: 0.73, and O: 0.74
Hassanzadeh et al. (2014) [20]	PIBOSO (Population-Intervention-Background-Outcome-Study Design-Other)	PIBOSO corpus, 1000 structured and unstructured abstracts	CRF with discriminate set of features	Sentence	Abstract	Micro-averaged F-score: 91
Robinson (2012) [21]	Patient-oriented evidence: morbidity, morality, symptom severity, quality of life	1356 PubMed abstracts	SVM, NB, multinomial NB, logistic regression	Sentence	Abstract	Best results achieved via SVM: F-measure of 0.86
Chung (2009) [22]	Intervention, comparisons	203 RCT abstracts for training and 124 for testing	Coordinating constructs are identified using a full parser, which are further classified as positive or not using CRF	Sentence	Abstract	F-score: 0.76
Hara and Matsumoto (2007) [23]	Patient population, comparison	200 abstracts labeled as ‘Neoplasms’ and ‘Clinical Trial, Phase III’	Categorizing noun phrases (NPs) into classes such as ‘Disease’, ‘Treatment’ etc. using CRF and use regular expressions on the sentence with classified Noun Phrases	Sentence	Abstract	F-measure of 0.91 for the task of noun phrase classification. Results of sentence classification: F-,measure of 0.8 for patient population and 0.81 for comparisons
Davis-Desmond and Molla (2012) [42]	Detecting statistical evidence	194 randomized controlled trial abstracts from PubMed	Rule-based classifier using negation expressions	Sentence	Abstract	Accuracy: between 88 and 98 % at 95 % CI
Zhao et al. (2012) [24]	Patient, result, Intervention, Study Design, Research Goal	19,893 medical abstracts and full text articles from 17 journal websites	Conditional random fields	Sentence	Full text	F-scores for sentence classification: patient: 0.75, intervention: 0.61, result: 0.91, study design: 0.79, research goal: 0.76
Hsu et al. (2012) [25]	Hypothesis, statistical method, outcomes and generalizability	42 full-text papers	Regular expressions	Sentence	Full text	For classification task, F-score of 0.86 for hypothesis, 0.84 for statistical method, 0.9 for outcomes, and 0.59 for generalizability
Song et al. (2013) [26]	Analysis (statistical facts), general (generally accepted facts), recommend (recommendations about interventions), rule (guidelines)	346 sentences from three clinical guideline document	Maximum entropy (MaxEnt), SVM, MLP, radial basis function network (RBFN), NB as classifiers and information gain (IG), genetic algorithm (GA) for feature selection	Sentence	Full text	F-score of 0.98 for classifying sentences
Demner-Fushman and Lin (2007) [28]	PICO (I and C were combined)	275 manually annotated abstracts	Rule-based approach to identify sentence containing PICO and supervised classifier for Outcomes	Concept	Abstract	Precision of 0.8 for population, 0.86 for problem, 0.80 for intervention, 0.64–0.95 for outcome
Kelly and Yang (2013) [29]	Age of subjects, duration of study, ethnicity of subjects, gender of subjects, health status of subjects, number of subjects	386 abstracts from PubMed obtained with the query ‘soy and cancer’	Regular expressions, gazetteer	Concept	Abstract	F-scores for age of subjects: 1.0, duration of study: 0.911, ethnicity of subjects: 0.949, gender of subjects: 1.0, health status of subjects: 0.874, number of subjects: 0.963
Hansen et al. (2008) [30]	Number of trial participants	233 abstracts from PubMed	Support vector machines	Concept	Abstract	F-measure: 0.86
Xu et al. (2007) [32]	Subject demographics such as subject descriptors, number of participants and diseases/symptoms and their descriptors	250 randomized controlled trial abstracts	Text classification augmented with hidden Markov models was used to identify sentences; rules over parse tree to extract relevant information	Sentence, concept	Abstract	Precision for subject descriptors: 0.83 %, number of trial participants: 0.923, diseases/symptoms: 51.0 %, descriptors of diseases/symptoms: 92.0 %
Summerscales et al. (2009) [34]	Treatments, groups and outcomes	100 abstracts from *BMJ*	Conditional random fields	Concept	Abstract	F-scores for treatments: 0.49, groups: 0.82, outcomes: 0.54
Summerscales et al. (2011) [35]	Groups, outcomes, group sizes, outcome numbers	263 abstracts from *BMJ* between 2005 and 2009	CRF, MaxEnt, template filling	Concept	Abstract	F-scores for groups: 0.76, outcomes: 0.42, group sizes: 0.80, outcome numbers: 0.71
Kiritchenko et al. (2010) [36]	Eligibility criteria, sample size, drug dosage, primary outcomes	50 full-text journal articles with 1050 test instances	SVM classifier to recover relevant sentences, extraction rules for correct solutions	Concept	Full text	P5 precision for the classifier: 0.88, precision and recall of the extraction rules: 93 and 91 %, respectively
Lin et al. (2010) [39]	Intervention, age group of the patients, geographical area, number of patients, time duration of the study	93 open access full-text literature documenting oncological and cardio-vascular studies from 2005 to 2008	Linear chain, conditional random fields	Concept	Full text	Precision of 0.4 for intervention, 0.63 for age group, 0.44 for geographical area, 0.43 for number of patients and 0.83 for time period
Restificar et al. (2012) [37]	Eligibility criteria	44,203 full-text articles with clinical trials	Latent Dirichlet allocation along with logistic regression	Concept	Full text	75 and 70 % accuracy based on similarity for inclusion and exclusion criteria, respectively.
De Bruijn et al. (2008) [40]	Eligibility criteria, sample size, treatment duration, intervention, primary and secondary outcomes	88 randomized controlled trials full-text articles from five medical journals	SVM classifier to identify the most promising sentences; manually crafted weak extraction rules for the information elements	Sentence, concept	Full text	Precision for eligibility criteria: 0.69, sample size: 0.62, treatment duration: 0.94, intervention: 0.67, primary outcome: 1.00, secondary outcome: 0.67
Zhu et al. (2012) [41]	Subject demographics: patient age, gender, disease and ethnicity	50 randomized controlled trials full-text articles	Manually crafted rules for extraction from the parse tree	Concept	Full text	Disease extraction: for exact matching, the F-score was 0.64. For partially matched, it was 0.85.
Marshall et al. (2014) [27]	Risk of bias concerning sequence generation, allocation concealment and blinding	2200 clinical trial reports	Soft-margin SVM for a joint model of risk of bias prediction and supporting sentence extraction	Sentence	Full text	For sentence identification: F-score of 0.56, 0.48, 0.35 and 0.38 for random sequence generation, allocation concealment, blinding of participants and personnel, and blinding of outcome assessment

The accuracy of most (*N* = 18, 69 %) studies was measured using a standard text mining metric known as F-score, which is the harmonic mean of precision (positive predictive value) and recall (sensitivity). Some studies (*N* = 5, 19 %) reported only the precision of their method, while some reported the accuracy values (*N* = 2, 8 %). One study (4 %) reported P5 precision, which indicates the fraction of positive predictions among the top 5 results returned by the system.

### Studies that did not implement a data extraction system

Dawes et al. [12] identified 20 evidence-based medicine journal synopses with 759 extracts in the corresponding PubMed abstracts. Annotators agreed with the identification of an element 85 and 87 % for the evidence-based medicine synopses and PubMed abstracts, respectively. After consensus among the annotators, agreement rose to 97 and 98 %, respectively. The authors proposed various lexical patterns and developed rules to discover each PECODR element from the PubMed abstracts and the corresponding evidence-based medicine journal synopses that might make it possible to partially or fully automate the data extraction process.

### Studies that identified sentences but did not extract data elements from abstracts only

Kim et al. [13] used conditional random fields (CRF) [15] for the task of classifying sentences in one of the PICO categories. The features were based on lexical, syntactic, structural, and sequential information in the data. The authors found that unigrams, section headings, and sequential information from preceding sentences were useful features for the classification task. They used 1000 medical abstracts from PIBOSO corpus and achieved micro-averaged F-scores of 91 and 67 % over datasets of structured and unstructured abstracts, respectively.

Boudin et al. [16] utilized a combination of multiple supervised classification techniques for detecting PICO elements in the medical abstracts. They utilized features such as MeSH semantic types, word overlap with title, number of punctuation marks on random forests (RF), naive Bayes (NB), support vector machines (SVM), and multi-layer perceptron (MLP) classifiers. Using 26,000 abstracts from PubMed, the authors took the first sentence in the structured abstracts and assigned a label automatically to build a large training data. They obtained an F-score of 86 % for identifying participants (P), 67 % for interventions (I) and controls (C), and 56 % for outcomes (O).

Huang et al. [17] used a naive Bayes classifier for the PICO classification task. The training data were generated automatically from the structured abstracts. For instance, all sentences in the section of the structured abstract that started with the term “PATIENT” were used to identify participants (P). In this way, the authors could generate a dataset of 23,472 sentences. Using 23,472 sentences from the structured abstracts, they obtained an F-score of 91 % for identifying participants (P), 75 % for interventions (I), and 88 % for outcomes (O).

Verbeke et al. [18] used a statistical relational learning-based approach (kLog) that utilized relational features for classifying sentences. The authors also used the PIBOSO corpus for evaluation and achieved micro-averaged F-score of 84 % on structured abstracts and 67 % on unstructured abstracts, which was a better performance than Kim et al. [13].

Huang et al. [19] used 19,854 structured extracts and trained two classifiers: one by taking the first sentences of each section (termed CF by the authors) and the other by taking all the sentences in each section (termed CA by the authors). The authors used the naive Bayes classifier and achieved F-scores of 74, 66, and 73 % for identifying participants (P), interventions (I), and outcomes (O), respectively, by the CF classifier. The CA classifier gave F-scores of 73, 73, and 74 % for identifying participants (P), interventions (I), and outcomes (O), respectively.

Hassanzadeh et al. [20] used the PIBOSO corpus for the identification of sentences with PIBOSO elements. Using conditional random fields (CRF) with discriminative set of features, they achieved micro-averaged F-score of 91 %.

Robinson [21] used four machine learning models, 1) support vector machines, 2) naive Bayes, 3) naive Bayes multinomial, and 4) logistic regression to identify medical abstracts that contained patient-oriented evidence or not. These data included morbidity, mortality, symptom severity, and health-related quality of life. On a dataset of 1356 PubMed abstracts, the authors achieved the highest accuracy using a support vector machines learning model and achieved an F-measure of 86 %.

Chung [22] utilized a full sentence parser to identify the descriptions of the assignment of treatment arms in clinical trials. The authors used predicate-argument structure along with other linguistic features with a maximum entropy classifier. They utilized 203 abstracts from randomized trials for training and 124 abstracts for testing and achieved an F-score of 76 %.

Hara and Matsumoto [23] dealt with the problem of extracting “patient population” and “compared treatments” from medical abstracts. Given a sentence from the abstract, the authors first performed base noun-phrase chunking and then categorized the base noun-phrase into one of the five classes: “disease”, “treatment”, “patient”, “study”, and “others” using support vector machine and conditional random field models. After categorization, the authors used regular expression to extract the target words for patient population and comparison. The authors used 200 abstracts including terms such as “neoplasms” and “clinical trial, phase III” and obtained 91 % accuracy for the task of noun phrase classification. For sentence classification, the authors obtained a precision of 80 % for patient population and 82 % for comparisons.

### Studies that identified only sentences but did not extract data elements from full-text reports

Zhao et al. [24] used two classification tasks to extract study data including patient details, including one at the sentence level and another at the keyword level. The authors first used a five-class scheme including 1) patient, 2) result, 3) intervention, 4) study design, and 5) research goal and tried to classify sentences into one of these five classes. They further used six classes for keywords such as sex (e.g., male, female), age (e.g., 54-year-old), race (e.g., Chinese), condition (e.g., asthma), intervention, and study design (e.g., randomized trial). They utilized conditional random fields for the classification task. Using 19,893 medical abstracts and full-text articles from 17 journal websites, they achieved F-scores of 75 % for identifying patients, 61 % for intervention, 91 % for results, 79 % for study design, and 76 % for research goal.

Hsu et al. [25] attempted to classify whether a sentence contains the “hypothesis”, “statistical method”, “outcomes”, or “generalizability” of the study and then extracted the values. Using 42 full-text papers, the authors obtained F-scores of 86 % for identifying hypothesis, 84 % for statistical method, 90 % for outcomes, and 59 % for generalizability.

Song et al. [26] used machine learning-based classifiers such as maximum entropy classifier (MaxEnt), support vector machines (SVM), multi-layer perceptron (MLP), naive Bayes (NB), and radial basis function network (RBFN) to classify the sentences into categories such as analysis (statistical facts found by clinical experiment), general (generally accepted scientific facts, process, and methodology), recommendation (recommendations about interventions), and rule (guidelines). They utilized the principle of information gain (IG) as well as genetic algorithm (GA) for feature selection. They used 346 sentences from the clinical guideline document and obtained an F-score of 98 % for classifying sentences.

Marshall et al. [27] used soft-margin support vector machines in a joint model for risk of bias assessment along with supporting sentences for random sequence generation, allocation concealment, blinding of participants and personnel, and blinding of outcome assessment, among others. They utilized presence of unigrams in the supporting sentences as features in their model. Working with full text of 2200 clinical trials, the joint model achieved F-scores of 56, 48, 35, and 38 % for identifying sentences corresponding to random sequence generation, allocation concealment, blinding of participants and personnel, and blinding of outcome assessment, respectively.

### Studies that identified data elements only from abstracts but not from full texts

Demner-Fushman and Lin [28] used a rule-based approach to identify sentences containing PICO. Using 275 manually annotated abstracts, the authors achieved an accuracy of 80 % for population extraction and 86 % for problem extraction. They also utilized a supervised classifier for outcome extraction and achieved accuracy from 64 to 95 % across various experiments.

Kelly and Yang [29] used regular expressions and gazetteer to extract the number of participants, participant age, gender, ethnicity, and study characteristics. The authors utilized 386 abstracts from PubMed obtained with the query “soy and cancer” and achieved F-scores of 96 % for identifying the number of participants, 100 % for age of participants, 100 % for gender of participants, 95 % for ethnicity of participants, 91 % for duration of study, and 87 % for health status of participants.

Hansen et al. [30] used support vector machines [31] to extract number of trial participants from abstracts of the randomized control trials. The authors utilized features such as part-of-speech tag of the previous and next words and whether the sentence is grammatically complete (contained a verb). Using 233 abstracts from PubMed, they achieved an F-score of 86 % for identifying participants.

Xu et al. [32] utilized text classifications augmented with hidden Markov models [33] to identify sentences about subject demographics. These sentences were then parsed to extract information regarding participant descriptors (e.g., men, healthy, elderly), number of trial participants, disease/symptom name, and disease/symptom descriptors. After testing over 250 RCT abstracts, the authors obtained an accuracy of 83 % for participant descriptors: 83 %, 93 % for number of trial participants, 51 % for diseases/symptoms, and 92 % for descriptors of diseases/symptoms.

Summerscales et al. [34] used a conditional random field-based approach to identify various named entities such as treatments (drug names or complex phrases) and outcomes. The authors extracted 100 abstracts of randomized trials from the *BMJ* and achieved F-scores of 49 % for identifying treatment, 82 % for groups, and 54 % for outcomes.

Summerscales et al. [35] also proposed a method for automatic summarization of results from the clinical trials. The authors first identified the sentences that contained at least one integer (group size, outcome numbers, etc.). They then used the conditional random field classifier to find the entity mentions corresponding to treatment groups or outcomes. The treatment groups, outcomes, etc. were then treated as various “events.” To identify all the relevant information for these events, the authors utilized templates with slots. The slots were then filled using a maximum entropy classifier. They utilized 263 abstracts from the *BMJ* and achieved F-scores of 76 % for identifying groups, 42 % for outcomes, 80 % for group sizes, and 71 % for outcome numbers.

### Studies that identified data elements from full-text reports

Kiritchenko et al. [36] developed ExaCT, a tool that assists users with locating and extracting key trial characteristics such as eligibility criteria, sample size, drug dosage, and primary outcomes from full-text journal articles. The authors utilized a text classifier in the first stage to recover the relevant sentences. In the next stage, they utilized extraction rules to find the correct solutions. The authors evaluated their system using 50 full-text articles describing randomized trials with 1050 test instances and achieved a P5 precision of 88 % for identifying the classifier. Precision and recall of their extraction rules was found to be 93 and 91 %, respectively.

Restificar et al. [37] utilized latent Dirichlet allocation [38] to infer the latent topics in the sample documents and then used logistic regression to compute the probability that a given candidate criterion belongs to a particular topic. Using 44,203 full-text reports of randomized trials, the authors achieved accuracies of 75 and 70 % for inclusion and exclusion criteria, respectively.

Lin et al. [39] used linear-chain conditional random field for extracting various metadata elements such as number of patients, age group of the patients, geographical area, intervention, and time duration of the study. Using 93 full-text articles, the authors achieved a threefold cross validation precision of 43 % for identifying number of patients, 63 % for age group, 44 % for geographical area, 40 % for intervention, and 83 % for time period.

De Bruijn et al. [40] used support vector machine classifier to first identify sentences describing information elements such as eligibility criteria, sample size, etc. The authors then used manually crafted weak extraction rules to extract various information elements. Testing this two-stage architecture on 88 randomized trial reports, they obtained a precision of 69 % for identifying eligibility criteria, 62 % for sample size, 94 % for treatment duration, 67 % for intervention, 100 % for primary outcome estimates, and 67 % for secondary outcomes.

Zhu et al. [41] also used manually crafted rules to extract various subject demographics such as disease, age, gender, and ethnicity. The authors tested their method on 50 articles and for disease extraction obtained an F-score of 64 and 85 % for exactly matched and partially matched cases, respectively.

### Risk of bias across studies

In general, many studies have a high risk of selection bias because the gold standards used in the respective studies were not randomly selected. The risk of performance bias is also likely to be high because the investigators were not blinded. For the systems that used rule-based approaches, it was unclear whether the gold standard was used to train the rules or if there were a separate training set. The risk of attrition bias is unclear based on the study design of these non-randomized studies evaluating the performance of NLP methods. Lastly, the risk of reporting bias is unclear because of the lack of protocols in the development, implementation, and evaluation of NLP methods.

## Discussion

### Summary of evidence

#### Extracting the data elements

- Participants**—**Sixteen studies explored the extraction of the number of participants [12, 13, 16–20, 23, 24, 28–30, 32, 39], their age [24, 29, 39, 41], sex [24, 39], ethnicity [41], country [24, 39], comorbidities [21], spectrum of presenting symptoms, current treatments, and recruiting centers [21, 24, 28, 29, 32, 41], and date of study [39]. Among them, only six studies [28–30, 32, 39, 41] extracted data elements as opposed to highlighting the sentence containing the data element. Unfortunately, each of these studies used a different corpus of reports, which makes direct comparisons impossible. For example, Kelly and Yang [29] achieved high F-scores of 100 % for age of participants, 91 % for duration of study, 95 % for ethnicity of participants, 100 % for gender of subjects, 87 % for health status of participants, and 96 % for number of participants on a dataset of 386 abstracts.
- Intervention**—**Thirteen studies explored the extraction of interventions [12, 13, 16–20, 22, 24, 28, 34, 39, 40], intervention groups [34, 35], and intervention details (for replication if feasible) [36]. Of these, only six studies [28, 34–36, 39, 40] extracted intervention elements. Unfortunately again, each of these studies used a different corpus. For example, Kiritchenko et al. [36] achieved an F-score of 75–86 % for intervention data elements on a dataset of 50 full-text journal articles.
- Outcomes and comparisons**—**Fourteen studies also explored the extraction of outcomes and time points of collection and reporting [12, 13, 16–20, 24, 25, 28, 34–36, 40] and extraction of comparisons [12, 16, 22, 23]. Of these, only six studies [28, 34–36, 40] extracted the actual data elements. For example, De Bruijn et al. [40] obtained an F-score of 100 % for extracting primary outcome and 67 % for secondary outcome from 88 full-text articles. Summerscales [35] utilized 263 abstracts from the *BMJ* and achieved an F-score of 42 % for extracting outcomes.
- Results**—**Two studies [36, 40] extracted sample size data element from full text on two different data sets. De Bruijn et al. [40] obtained an accuracy of 67 %, and Kiritchenko et al. [36] achieved an F-score of 88 %.
- Interpretation**—**Three studies explored extraction of overall evidence [26, 42] and external validity of trial findings [25]. However, all these studies only highlighted sentences containing the data elements relevant to interpretation.
- Objectives**—**Two studies [24, 25] explored the extraction of research questions and hypotheses. However, both these studies only highlighted sentences containing the data elements relevant to interpretation.
- Methods**—**Twelve studies explored the extraction of the study design [13, 18, 20, 24], study duration [12, 29, 40], randomization method [25], participant flow [36, 37, 40], and risk of bias assessment [27]. Of these, only four studies [29, 36, 37, 40] extracted the corresponding data elements from text using different sets of corpora. For example, Restificar et al. [37] utilized 44,203 full-text clinical trial articles and achieved accuracies of 75 and 70 % for inclusion and exclusion criteria, respectively.
- Miscellaneous**—**One study [26] explored extraction of key conclusion sentence and achieved a high F-score of 98 %.

### Related reviews and studies

Previous reviews on the automation of systematic review processes describe technologies for automating the overall process or other steps. Tsafnat et al. [43] surveyed the informatics systems that automate some of the tasks of systematic review and report systems for each stage of systematic review. Here, we focus on data extraction. None of the existing reviews [43–47] focus on the data extraction step. For example, Tsafnat et al. [43] presented a review of techniques to automate various aspects of systematic reviews, and while data extraction has been described as a task in their review, they only highlighted three studies as an acknowledgement of the ongoing work. In comparison, we identified 26 studies and critically examined their contribution in relation to all the data elements that need to be extracted to fully support the data extraction step.

Thomas et al. [44] described the application of text mining technologies such as automatic term recognition, document clustering, classification, and summarization to support the identification of relevant studies in systematic reviews. The authors also pointed out the potential of these technologies to assist at various stages of the systematic review. Slaughter et al. [45] discussed necessary next steps towards developing “living systematic reviews” rather than a static publication, where the systematic reviews can be continuously updated with the latest knowledge available. The authors mentioned the need for development of new tools for reporting on and searching for structured data from clinical trials.

Tsafnat et al. [46] described four main tasks in systematic review: identifying the relevant studies, evaluating risk of bias in selected trials, synthesis of the evidence, and publishing the systematic reviews by generating human-readable text from trial reports. They mentioned text extraction algorithms for evaluating risk of bias and evidence synthesis but remain limited to one particular method for extraction of PICO elements.

Most natural language processing research has focused on reducing the workload for the screening step of systematic reviews (Step 3). Wallace et al. [48, 49] and Miwa et al. [50] proposed an active learning framework to reduce the workload in citation screening for inclusion in the systematic reviews. Jonnalagadda et al. [51] designed a distributional semantics-based relevance feedback model to semi-automatically screen citations. Cohen et al. [52] proposed a module for grouping studies that are closely related and an automated system to rank publications according to the likelihood for meeting the inclusion criteria of a systematic review. Choong et al. [53] proposed an automated method for automatic citation snowballing to recursively pursue relevant literature for helping in evidence retrieval for systematic reviews. Cohen et al. [54] constructed a voting perceptron-based automated citation classification system to classify each article as to whether it contains high-quality, drug-specific evidence. Adeva et al. [55] also proposed a classification system for screening articles for systematic review. Shemilt et al. [56] also discussed the use of text mining to reduce screening workload in systematic reviews.

### Research implications

#### No standard gold standards or dataset

Among the 26 studies included in this systematic review, only three of them use a common corpus, namely 1000 medical abstracts from the PIBOSO corpus. Unfortunately, even that corpus facilitates only classification of sentences into whether they contain one of the data elements corresponding to the PIBOSO categories. No two other studies shared the same gold standard or dataset for evaluation. This limitation made it impossible for us to compare and assess the relative significance of the reported accuracy measures.

### Separate systems for each data element

Few data elements, which are also relatively straightforward to extract automatically, such as the total number of participants (14 overall and 5 for extracting the actual data elements), have a relatively higher number of studies aiming towards extracting the same data element. This is not the case with other data elements. There are 27 out of 52 potential data elements that have not been explored for automated extraction, even if for highlighting the sentences containing them; seven more data elements were explored just by one study. There are 38 out of 52 potential data elements (>70 %) that have not been explored for automated extraction of the actual data elements; three more data elements were explored just by one study. The highest number of data elements extracted by a single study is only seven (14 %). This finding means that not only are more studies needed to explore the remaining 70 % data elements, but that there is an urgent need for a unified framework or system to extract all necessary data elements. The current state of informatics research for data extraction is exploratory, and multiple studies need to be conducted using the same gold standard and on the extraction of the same data elements for effective comparison.

### Limitations

Our study has limitations. First, there is a possibility that data extraction algorithms were not published in journals or that our search might have missed them. We sought to minimize this limitation by searching in multiple bibliographic databases, including PubMed, IEEExplore, and ACM Digital Library. However, investigators may have also failed to publish algorithms that had lower F-scores than were previously reported, which we would not have captured. Second, we did not publish a protocol a priori, and our initial findings may have influenced our methods. However, we performed key steps, including screening, full-text review, and data extraction in duplicate to minimize potential bias in our systematic review.

### Future work

“On demand” access to summarized evidence and best practices has been considered a sound strategy to satisfy clinicians’ information needs and enhance decision-making [57–65]. A systematic review of 26 studies concluded that information-retrieval technology produces positive impact on physicians in terms of decision enhancement, learning, recall, reassurance, and confirmation [62]. Slaughter et al. [45] discussed necessary next steps towards developing “living systematic reviews” rather than a static publication, where the systematic reviews can be continuously updated with the latest knowledge available. The authors mention the need for development of new tools for reporting on and searching for structured data from published literature. Automated information extraction framework that extract data elements have the potential to assist the systematic reviewers and to eventually automate the screening and data extraction steps.

Medical science is currently witnessing a rapid pace at which medical knowledge is being created—75 clinical trials a day [66]. Evidence-based medicine [67] requires clinicians to keep up with published scientific studies and use them at the point of care. However, it has been shown that it is practically impossible to do that even within a narrow specialty [68]. A critical barrier is that finding relevant information, which may be located in several documents, takes an amount of time and cognitive effort that is incompatible with the busy clinical workflow [69, 70]. Rapid systematic reviews using automation technologies will enable clinicians with up-to-date and systematic summaries of the latest evidence.

## Conclusions

Our systematic review describes previously reported methods to identify sentences containing some of the data elements for systematic reviews and only a few studies that have reported methods to extract these data elements. However, most of the data elements that would need to be considered for systematic reviews have been insufficiently explored to date, which identifies a major scope for future work. We hope that these automated extraction approaches might first act as checks for manual data extraction currently performed in duplicate; then serve to validate manual data extraction done by a single reviewer; then become the primary source for data element extraction that would be validated by a human; and eventually completely automate data extraction to enable living systematic reviews.

## Appendix 1

### Search strategies

Below, we provide the search strategies used in PubMed, ACM Digital Library, and IEEExplore. The search was conducted on January 6, 2015.

### PubMed

(“identification” [Title] OR “extraction” [Title] OR “extracting” [Title] OR “detection” [Title] OR “identifying” [Title] OR “summarization” [Title] OR “learning approach” [Title] OR “automatically” [Title] OR “summarization” [Title] OR “identify sections” [Title] OR “learning algorithms” [Title] OR “Interpreting” [Title] OR “Inferring” [Title] OR “Finding” [Title] OR “classification” [Title]) AND (“medical evidence”[Title] OR “PICO”[Title] OR “PECODR” [Title] OR “intervention arms” [Title] OR “experimental methods” [Title] OR “study design parameters” [Title] OR “Patient oriented Evidence” [Title] OR “eligibility criteria” [Title] OR “clinical trial characteristics” [Title] OR “evidence based medicine” [Title] OR “clinically important elements” [Title] OR “evidence based practice” [Title] “results from clinical trials” [Title] OR “statistical analyses” [Title] OR “research results” [Title] OR “clinical evidence” [Title] OR “Meta Analysis” [Title] OR “Clinical Research” [Title] OR “medical abstracts” [Title] OR “clinical trial literature” [Title] OR ”clinical trial characteristics” [Title] OR “clinical trial protocols” [Title] OR “clinical practice guidelines” [Title]).

### IEEE

We performed this search only in the metadata.

(“identification” OR “extraction” OR “extracting” OR “detection” OR “Identifying” OR “summarization” OR “learning approach” OR “automatically” OR “summarization” OR “identify sections” OR “learning algorithms” OR “Interpreting” OR “Inferring” OR “Finding” OR “classification”) AND (“medical evidence” OR “PICO” OR “intervention arms” OR “experimental methods” OR “eligibility criteria” OR “clinical trial characteristics” OR “evidence based medicine” OR “clinically important elements” OR “results from clinical trials” OR “statistical analyses” OR “clinical evidence” OR “Meta Analysis” OR “clinical research” OR “medical abstracts” OR “clinical trial literature” OR “clinical trial protocols”).

### ACM digital library

((Title: “identification” or Title: “extraction” or Title: “extracting” or Title: “detection” or Title: “Identifying” or Title: “summarization” or Title: “learning approach” or Title: “automatically” or Title: “summarization “or Title: “identify sections” or Title: “learning algorithms” or Title: “scientific artefacts” or Title: “Interpreting” or Title: “Inferring” or Title: “Finding” or Title: “classification” or “statistical techniques”) and (Title: “medical evidence” or Abstract: “medical evidence” or Title: “PICO” or Abstract: “PICO” or Title: “intervention arms” or Title: “experimental methods” or Title: “study design parameters” or Title: “Patient oriented Evidence” or Abstract: “Patient oriented Evidence” or Title: “eligibility criteria” or Abstract: “eligibility criteria” or Title: “clinical trial characteristics” or Abstract: “clinical trial characteristics” or Title: “evidence based medicine” or Abstract: “evidence based medicine” or Title: “clinically important elements” or Title: “evidence based practice” or Title: “treatments” or Title: “groups” or Title: “outcomes” or Title: “results from clinical trials” or Title: “statistical analyses” or Abstract: “statistical analyses” or Title: “research results” or Title: “clinical evidence” or Abstract: “clinical evidence” or Title: “Meta Analysis” or Abstract:“Meta Analysis” or Title:“Clinical Research” or Title: “medical abstracts” or Title: “clinical trial literature” or Title: “Clinical Practice” or Title: “clinical trial protocols” or Abstract: “clinical trial protocols” or Title: “clinical questions” or Title: “clinical trial design”)).

## Appendix 2

**Table 3:** Checklist of items to consider in data collection or data extraction from Cochrane Handbook [1]

Source
• Study ID (created by review author)
• Report ID (created by review author)
• Review author ID (created by review author)
• Citation and contact details
Eligibility
• Confirm eligibility for review
• Reason for exclusion
Methods
• Study design
• Total study duration
• Sequence generation^a^
• Allocation sequence concealment^a^
• Blinding^a^
• Other concerns about bias^a^
Participants
• Total number
• Setting
• Diagnostic criteria
• Age
• Sex
• Country
• [Co-morbidity]
• [Socio-demographics]
• [Ethnicity]
• [Date of study]
Interventions
• Total number of intervention groups.
For each intervention and comparison group of interest:
• Specific intervention
• Intervention details (sufficient for replication, if feasible)
• [Integrity of intervention]
Outcomes
• Outcomes and time points (i) collected; (ii) reported^a^
For each outcome of interest:
• Outcome definition (with diagnostic criteria if relevant)
• Unit of measurement (if relevant)
• For scales: upper and lower limits, and whether high or low score is good
Results
• Number of participants allocated to each intervention group.
For each outcome of interest:
• Sample size
• Missing participants^a^
• Summary data for each intervention group (e.g. 2 × 2 table for dichotomous data; means and SDs for continuous data)
• [Estimate of effect with confidence interval; *P* value]
• [Subgroup analyses]
Miscellaneous
• Funding source
• Key conclusions of the study authors
• Miscellaneous comments from the study authors
• References to other relevant studies
• Correspondence required
• Miscellaneous comments by the review authors

Items without parentheses should normally be collected in all reviews; items in square brackets may be relevant to some reviews and not to others

^a^Full description required for standard items in the ‘Risk of bias’ tool

## Abbreviations

NLP: natural language processing
ONSORT: CONsolidated Standards Of Reporting Trials
STARD: Standards for Reporting of Diagnostic Accuracy
PICO: Population, Intervention, Comparison, Outcomes
PECODR: Patient-Population-Problem, Exposure-Intervention, Comparison, Outcome, Duration and Results
PIBOSO: Population, Intervention, Background, Outcome, Study Design, Other
CRF: conditional random fields
NB: naive Bayes
RCT: randomized control trial
BMJ: British Medical Journal
